# Identification and experimental verification of biomarkers related to butyrate metabolism in osteoarthritis

**DOI:** 10.1038/s41598-025-97346-z

**Published:** 2025-04-07

**Authors:** Yi Zhang, Youliang Shen, Dewei Kou, Tengbo Yu

**Affiliations:** 1 Department of Orthopedics, Affiliated Hospital of Qingdao University, 16 Jiangsu Road, Shinan District, QingDao, 266003 China; 2 Traumatic Orthopedics Institute of Shandong, Affiliated Hospital of Qingdao University, Qingdao, China; 3 Department of Joint Surgery, Affiliated Hospital of Qingdao University, QingDao, China; 4 Department of Pain Management, Affiliated Hospital of Qingdao University, QingDao, China; 5 Department of Orthopedics, Qingdao Municipal Hospital, QingDao, China

**Keywords:** Osteoarthritis, Butyrate metabolism-related genes, Biomarkers, Bioinformatics, Computational biology and bioinformatics, Machine learning, Osteoarthritis, Bioinformatics

## Abstract

Butyrate plays a crucial role in osteoarthritis (OA) development. However, the relationship between butyrate metabolism-related genes (BMRGs) and OA remains unclear. This study investigates the potential correlation between BMRGs and OA using OA-related datasets (GSE55235, GSE12021 and GSE143514). Differential expression analysis identified 38 differentially expressed butyrate metabolism-related genes (DE-BMRGs) from the overlap of 782 OA-related differentially expressed genes (DEGs) and 385 BMRGs in GSE55235. Enrichment analysis indicated that these DE-BMRGs were tightly associated with cell proliferation, differentiation, and apoptosis, which are key processes in OA pathogenesis. Six candidate biomarkers (IL1B, IGF1, CXCL8, PTGS2, SERPINE1, MMP9) were identified through two machine-learning algorithms. IL1B, CXCL8, and PTGS2 were upregulated in controls, exhibiting consistent patterns across validation datasets. Gene set enrichment analysis (GSEA) revealed that dysregulated expression of these biomarkers lead to abnormal cell proliferation and differentiation, contributing to OA progression. Furthermore, significant differences in immune cell infiltration—particularly activated and resting mast cells—along with correlations to immune regulatory factors (CD86, CXCL12, TNFSF9, IL6), highlighted potential therapeutic targets. Quantitative RT-PCR further confirmed elevated expression of IL1B, CXCL8 and PTGS2 in control group. This study identifies IL1B, CXCL8 and PTGS2 as OA-related biomarkers linked to butyrate metabolism, offering a theoretical foundation and potential therapeutic strategies.

**Supplementary Information:**

The online version contains supplementary material available at 10.1038/s41598-025-97346-z.

## Introduction

Osteoarthritis (OA) is a prevalent degenerative disorder of the orthopedic musculoskeletal system, characterized primarily by cartilage degeneration and periarticular bone hyperplasia. According to the World Health Organization, approximately 10% of men, 18% of women, and 60–65% of the global population—over 300 million individuals—are affected by symptomatic OA, with 80% of these patients experiencing mobility impairments^1^. OA leads to structural damage to cartilage and subchondral bone, joint deformities, and motor dysfunction. The disease can involve nearly all joint tissues, including hyaline articular cartilage, subchondral bone, synovial membrane, and soft tissue such as ligaments, muscles, and meniscus, resulting in clinical symptoms such as pain, mobility limitations, joint stiffness, and disturbances in mood and sleep^2^. While the exact pathogenic mechanisms of OA remain incompletely understood, it is generally believed to arise from a multifactorial process involving obesity, aging, trauma, joint overuse, metabolic dysfunction, inflammation, and genetic factors^3^. Currently, no interventions exist that can reverse the pathological course of OA, and surgical options like total knee arthroplasty remain the primary treatment for advanced stages^4^. Although biological therapies, including intra-articular stem cell injections, show promis^4^, the identification of effective biomarkers and their underlying mechanisms remains a significant challenge. Therefore, investigating novel biomarkers and their pathophysiology is critical for advancing OA treatment.

Butyrate, a short-chain fatty acid produced by the gut microbiome from undigested fiber metabolism in the large intestine, serves as a primary energy source for colonocytes, supporting intestinal homeostasis and function^5^. It is involved in epigenetic regulation *via*histone deacetylase^6^and NF-κB signaling^7^, as well as the activation of nuclear peroxisome proliferator-activated receptor^8^, modulating inflammation, pain, and lipid metabolism—key processes that likely play significant roles in OA pathogenesis. The gut microbiome has recently been identified as a key factor in OA initiation and progression^9^. Studies have shown that OA is influenced by alterations in the gut microbiome, and butyrate may mitigate OA progression by modulating the gut environment^10,11^. Animal models has demonstrated that butyrate protects against OA, primarily by enhancing chondrocyte autophagic flux to reduce the production of inflammatory mediators^12,13^. Additionally, combined antibiotic and butyrate treatment alleviated cartilage degeneration in OA by decreasing lipopolysaccharide release, thus inhibiting lipopolysaccharide-induced inflammatory and improving gut microbiome dysbiosis^14^. However, the specific biomarkers and mechanisms through which butyrate metabolism influences OA progression remain unclear and warrant further investigation.

In the present study, OA-related transcriptomics data and butyrate metabolism-related genes (BMRGs) were analyzed through a series of bioinformatics approaches to evaluate the potential of BMRGs as biomarkers for OA, aiming to provid new insights for clinical diagnosis and treatment strategies.

## Results

### Identification and analysis of 38 differentially expressed (DE)-BMRGs

**Fig. 1:**
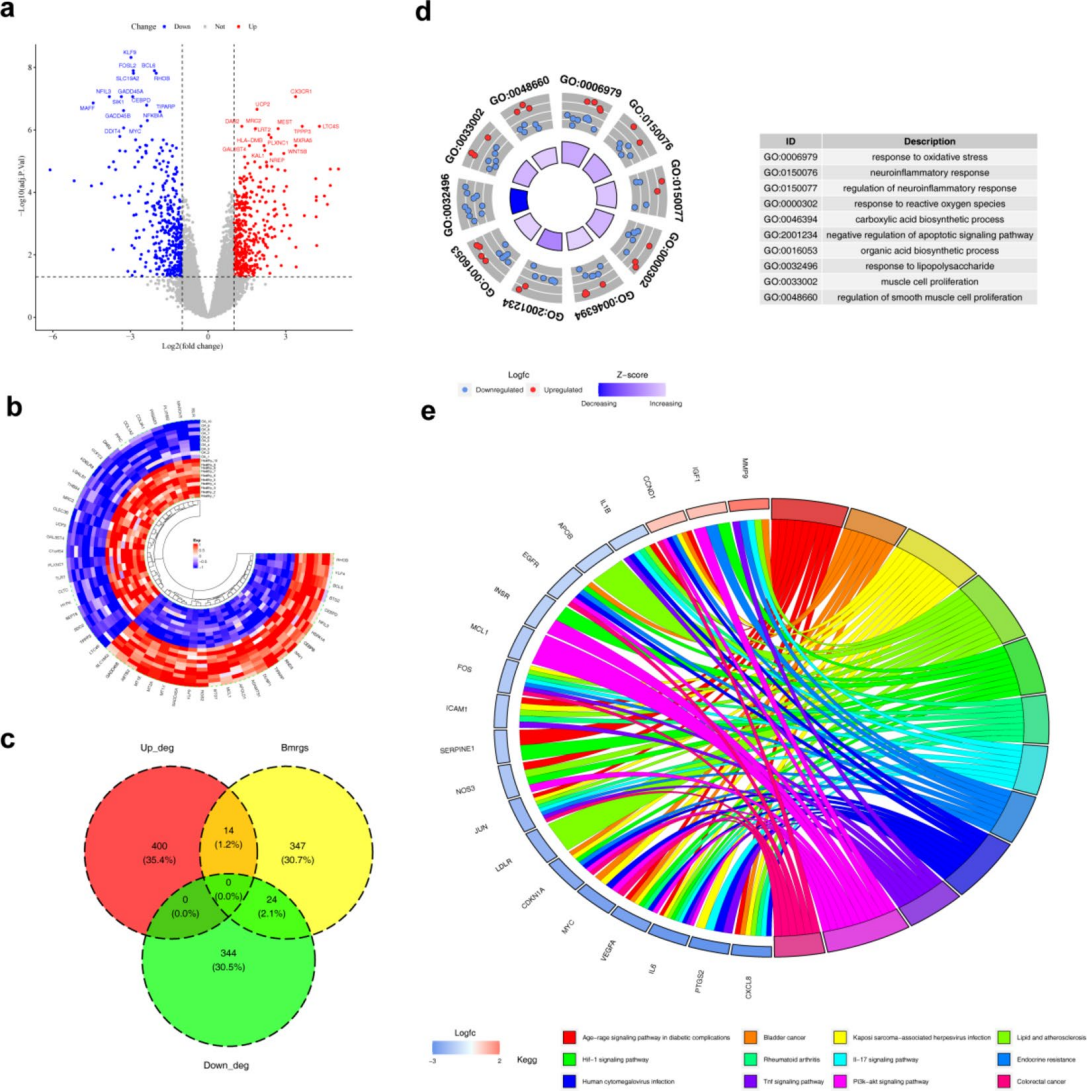
Diferentially expressed genes of osteoarthritis and BMRGs. (**a**) Volcano plot for the DEGs. Red and blue plot triangles represent upregulated and downregulated DEGs, respectively, with genes showing no significant differences indicated in gray. (**b**) Heatmap of DEGs. Red denotes high expression, and blue denotes low expression. (**c**) Venn diagram illustrating the overlap between OA DEGs and BMRGs. (**d**) GO analysis results for the 38 DE-BMRGs. (**e**) KEGG pathway analysis results. Different pathways are color-coded for distinction. DEGs, diferentially expressed genes.

Differential expression analysis identified 782 differentially expressed genes (DEGs) between OA and control groups in the GSE55235 dataset. Of these, 414 genes were upregulated and 364 downregulated in the OA group. The top10 DEGs are displayed in Fig. 1a and b. By intersecting the 782 DEGs and 385 BMRGs, 38 differentially expressed BMRGs (DE-BMRGs) were selected for further investigation **(**Fig. 1c**)**. Subsequent enrichment analyses revealed 1,528 Gene Ontology (GO) terms and 105 Kyoto Encyclopedia of Genes and Genomes (KEGG) pathways significantly enriched by DE-BMRGs. In GO analysis, DE-BMRGs were primarily associated with muscle cell proliferation-related functions and various response processes, including response to oxidative stress, reactive oxygen species, and neuroinflammatory responses **(**Fig. 1d**)** (*p*.adjust < 0.05). KEGG pathway analysis highlighted several cancer-related pathways, including the PI3 K-Akt and HIF- 1 signaling pathways, enriched by DE-BMRGs **(**Fig. 1e**)** (*p* < 0.05). These pathways, linked to cell proliferation, differentiation, and apoptosis, may contribute to the onset and progression of OA.

## IL1B, CXCL8, and PTGS2 were identified as biomarkers in OA

**Fig. 2:**
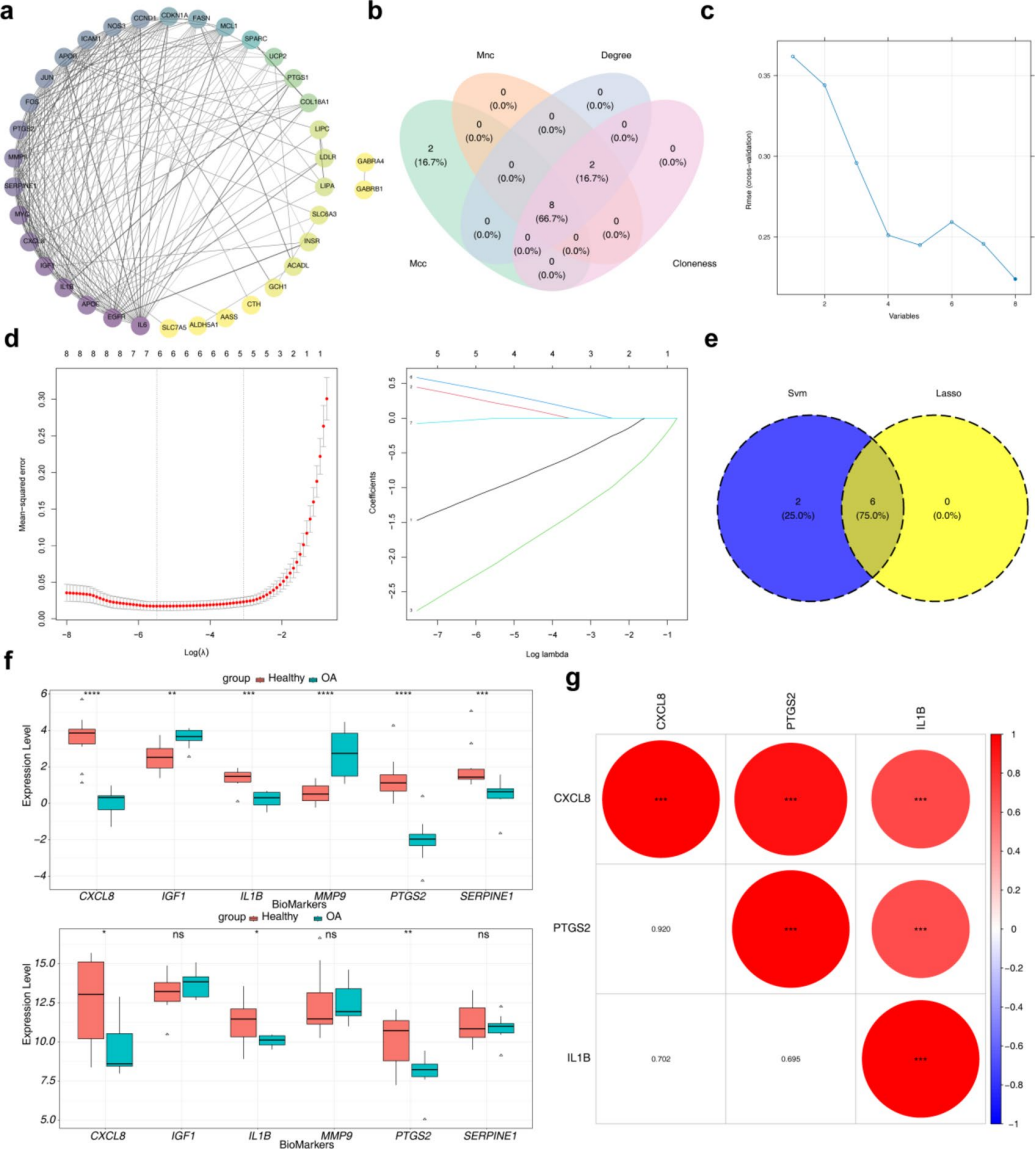
Protein interaction analysis in biological systems. (**a**) Protein–Protein Interaction (PPI) Networks visualization. (**b**) Venn diagram of the key genes identified by four algorithms. (**c**) Hub genes screening using the Support Vector Machine (SVM) algorithm. (**d**− 1) Lasso coefficient path results. (**d**− 2) Cross-validation curve results. (**e**) Candidate biomarkers identified by two machine learning algorithms. (**f**) Expression distribution of 6 candidate biomarkers in the training and validation sets. (**g**) Spearman correlation analysis of the 3 biomarkers in the validation set.

The protein–protein interaction (PPI) network of DE-BMRGs involved 36 nodes and 202 edges, with IL6 and EGFR showing the highest connectivity **(**Fig. 2a**)**. Intersection of the top 10 genes based on degree, MNC, closeness, and MCC scores revealed IL1B, IGF1, CXCL8, PTGS2, SERPINE1, MMP9, IL6, and EGFR as candidate hub genes **(**Fig. 2b**)**. These hub genes were further validated through two machine learning algorithms. All candidate hub genes were identified as signature genes by support vector machine-recursive feature elimination (SVM-RFE) **(**Fig. 2c**)**, while LASSO analysis (lambda.min = 0.004131806) selected six signature genes: IL1B, IGF1, CXCL8, PTGS2, SERPINE1, and MMP9 **(**Fig. 2d**)**. The intersection of these signature genes yielded six candidate biomarkers (IL1B, IGF1, CXCL8, PTGS2, SERPINE1, and MMP9) **(**Fig. 2e**)**. Among these, IL1B, CXCL8 and PTGS2 showed significant differential expression between OA and control groups, with higher expression in the control group in both the GSE55235 and GSE12021 datasets (*p* < 0.05) **(**Fig. 2f**)**. These three genes were identified as final biomarkers. Correlation analysis among the biomarkers revealed significant associations (|r| > 0.3, *p* < 0.05) **(**Fig. 2g**)**.

## The biomarkers were closely associated with OA

**Fig. 3:**
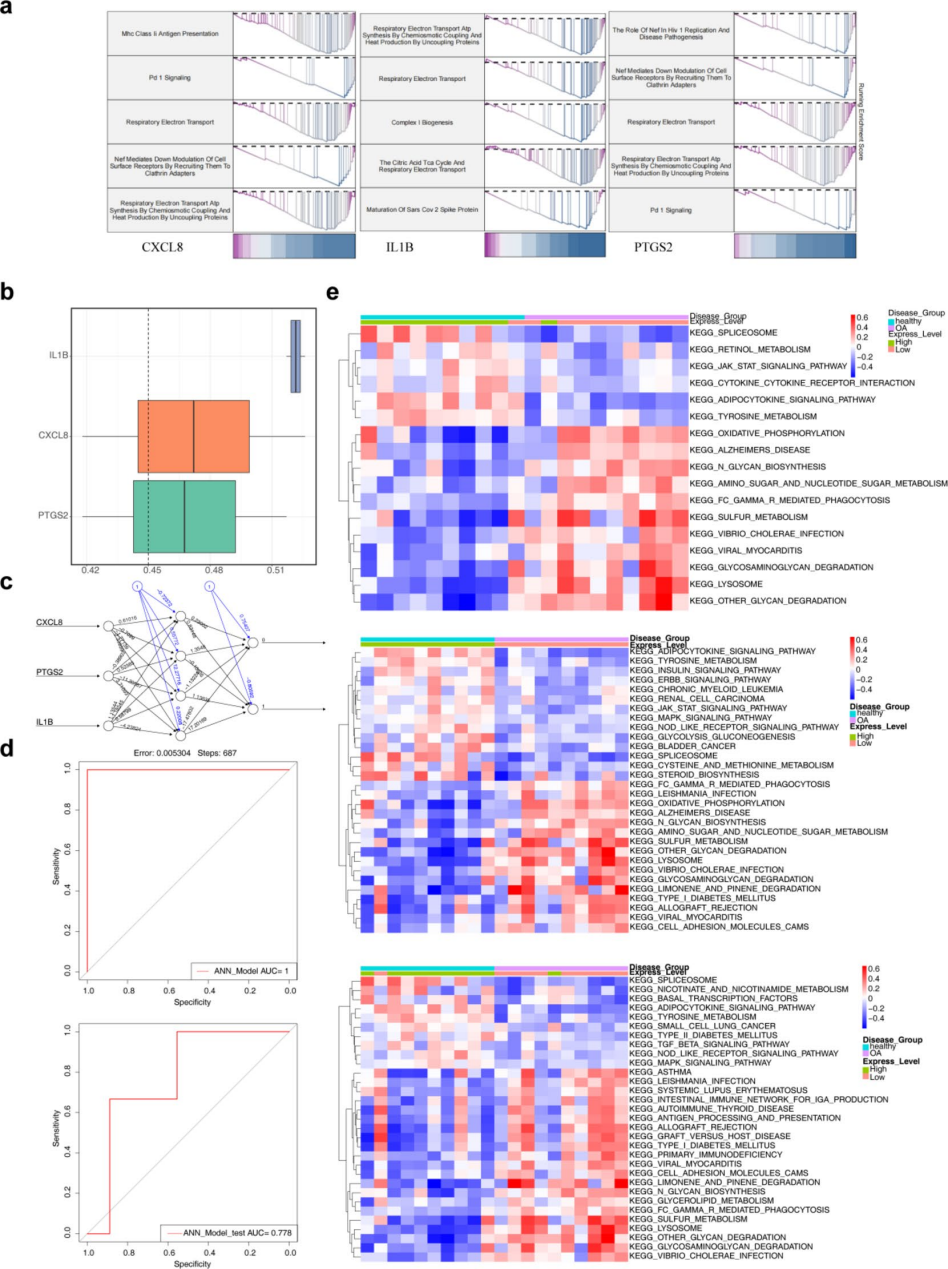
Gene set enrichment analysis (GSEA) for the biomarkers. (**a**) Gene Set Enrichment Analysis. The horizontal axis represents genes sorted by correlation, and the vertical axis indicates the enrichment pathway. (**b**) GOSemSim functional similarity analysis of the 3 biomarkers. (**c**) Artificial neural network (ANN) composed of the three biomarkers. (**d**) ROC curve evaluation for the ANN model in the training and validation sets. (**e**) GSVA pathway analysis of CXCL8, IL1B and PTGS2.

CXCL8, IL1B, and PTGS2 were enriched in 81, 76, and 68 gene sets, respectively. The top five reactome pathways with the lowest *p*-values were identified (*p*.adjust < 0.05, |NES| > 1) and are illustrated in Fig. 3a. These pathways are primarily associated with respiratory and immune processes, suggesting that aberrant biomarker expression may contribute to OA development. Functional similarity analysis revealed that CXCL8 exhibited similarities of 0.418 and 0.526 to other genes, IL1B showed similarities of 0.517 and 0.526, and PTGS2 displayed similarities of 0.418 and 0.517, indicating a high degree of similarity among the biomarkers **(**Fig. 3b**)**. An artificial neural network (ANN) model was constructed based on these biomarkers **(**Fig. 3c**)**, achieving an area under the curve (AUC) value greater than 0.7 in both the GSE55235 and GSE12021 datasets **(**Fig. 3d**)**. This result demonstrates the biomarkers’ potential diagnostic accuracy for OA. Gene set variation analysis (GSVA) revealed significant differences in 27, 17, and 31 pathways between high- and low-expression groups. Pathways related to cell proliferation, differentiation, oxidative phosphorylation metabolism, and immune responses were upregulated in the low-expression group, suggesting that reduced biomarker expression may disrupt energy metabolism, immune-inflammatory responses, and cell proliferation and differentiation **(**Fig. 3e**)**.

## The biomarkers interacted with multiple immune cell types to support OA progression

**Fig. 4:**
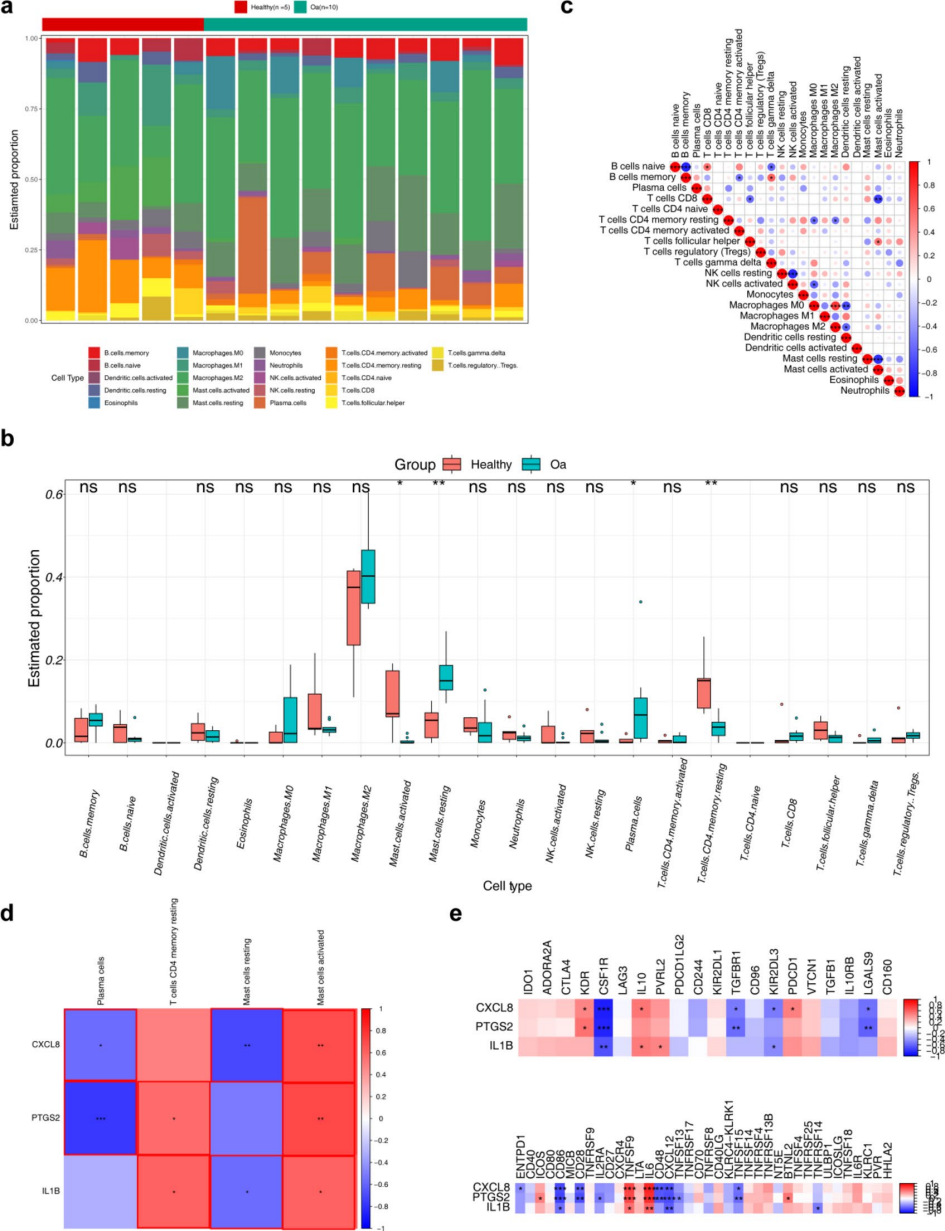
Immune infiltration analysis for the biomarkers. (**a**) Proportions of 22 immune cell types in two diferent samples visualized in a bar plot. (**b**) Comparison of immune cell content between OA and healthy groups. (**c**) Correlation of immune cells in OA and healthy samples from the training set. (**d**) Correlation between the biomarkers and the immune cells. A correlation coefficient |Cor| > 0.3 and *p* < 0.05 is marked as red. (**e**) Correlation between biomarkers and immunosuppressive factors. Red indicates positive correlation, and blue indicates negative correlation. *: *p* < 0.05; **: *p* < 0.01; ***: *p* < 0.001.

In the GSE55235 dataset, to ensure the reliability and validity of the immune infiltration analysis results, five control samples were excluded (*p* > 0.05), and the remaining samples were analysis. The compositional distribution of immune cells is shown in Fig. 4a. Among the 22 immune cell types, activated dendritic cells (DCs) and naïve CD4 T cells were absent. Significant differences were observed in activated and resting mast cells, plasma cells, and resting memory CD4 T cells. Activated mast cells and resting memory CD4 T cells were more abundant in the control group, while plasma cells and resting mast cells were elevated in the OA group **(**Fig. 4b**)**. Immune cell correlation analysis revealed a negative correlation between naive and memory B cells (*p* < 0.05) and a positive correlation between M0 and M2 macrophages **(**Fig. 4c**)**. Correlation analysis between biomarkers and differentially expressed immune cells showed that activated mast cells were positively correlated with biomarkers **(**Fig. 4d**)**. Furthermore, biomarkers exhibited a negative correlation with immune regulatory factors CD86 and CXCL12, and a positive correlation with TNFSF9 and IL6 (Fig. 4e). These immune regulatory factors may represent potential therapeutic targets for OA immune intervention.

## Biomarker mechanistic analysis

**Fig. 5:**
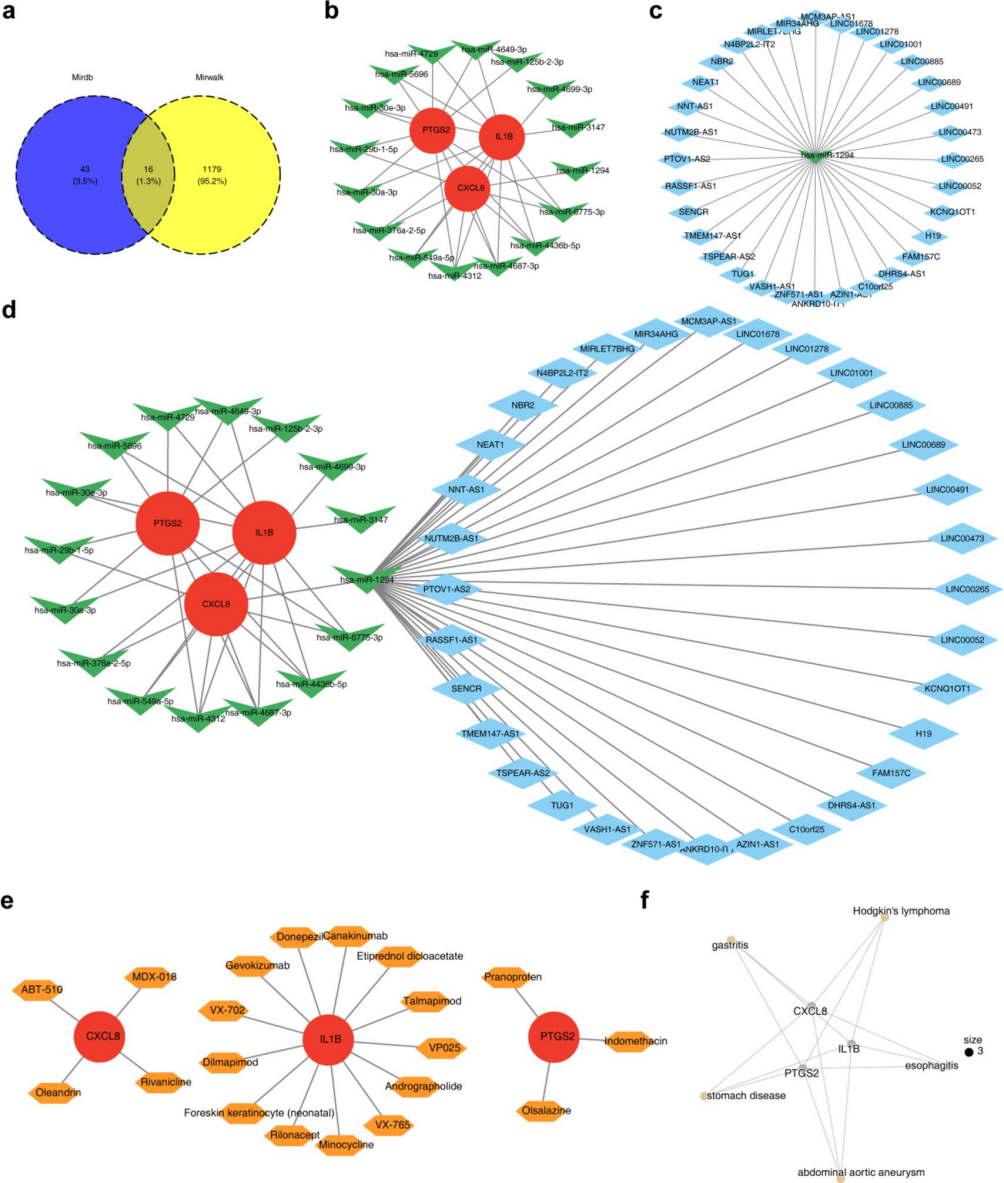
ceRNA regulatory network for the biomarkers. (**a**) Predicted shared miRNA for the biomarkers. (**b**) miRNA-mRNA regulatory network diagram. Green V shapes indicate miRNA, and red circles indicate biomarkers. (**c**) miRNA-IncRNA regulatory network diagram. Green V shapes indicate IncRNAs, and red circles indicate biomarkers. (**d**) ceRNA regulatory network diagram. Blue diamonds represent lncRNAs, green V shapes represent miRNA, and red ovals represent biomarkers. (**e**) Do analysis for the biomarkers. (**f**) Network graph for drug prediction analysis. Pink circles represent key genes, and orange hexagons represent drugs.

A total of 59 microRNAs (miRNAs) (target score ≥ 80) and 1,195 miRNAs (binding point = 1) were predicted using miRDB and miRWalk databases, respectively. By intersecting the predicted miRNAs, 16 key miRNA-targeting biomarkers were identified **(**Fig. 5a**)**. Additionally, 32 long non-coding RNA (lncRNA)-targeting key miRNAs were predicted using the miRNet database. Among these, hsa-miR- 4312, hsa-miR- 4687 - 3p, hsa-miR- 4436b- 5p, and hsa-miR- 6775b- 3p were found to regulate three biomarkers simultaneously **(**Fig. 5b**)**. All lncRNAs modulated CXCL8 *via* hsa-miR- 1294 **(**Fig. 5c and d**)**. In drug prediction analyses, Pranoprofen, Indomethacin, and Olsalazine were identified as targeting PTGS2, while ABT- 510, Oleandrin, MDX- 018, and Rivanicline focused on CXCL8. Additionally, 14 drugs, including Donepezil and Talmapimod, were predicted to target IL1B (Fig. 5e). Furthermore, 304 diseases were identified as being linked to these biomarkers, with the top five diseases—gastritis, stomach disease, Hodgkin’s lymphoma, esophagitis, and abdominal aortic aneurysm—highlighted in Fig. 5f.

### Validation of biomarker expression levels

**Fig. 6:**
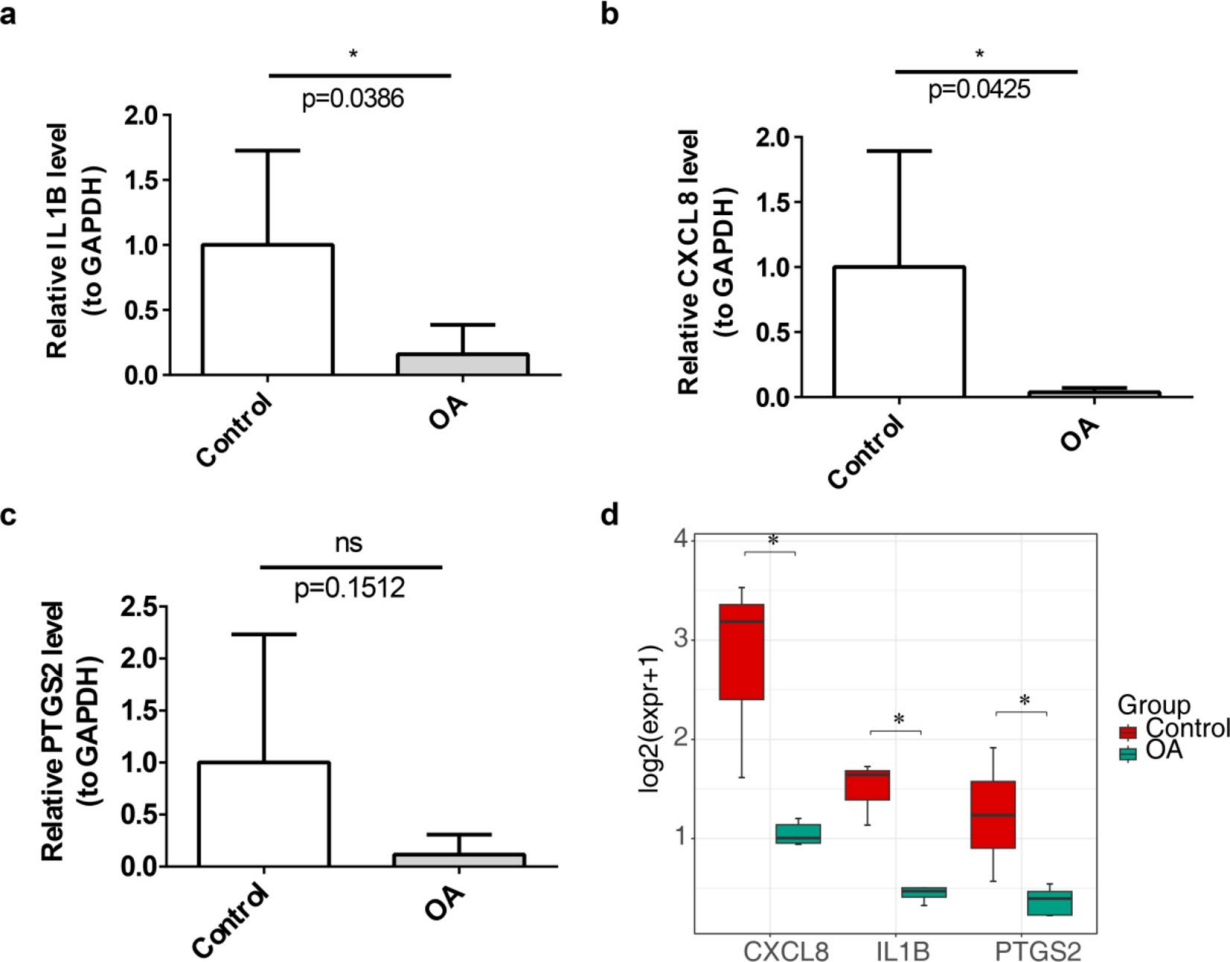
**Expression levels of three biomarkers. Differences in mRNA** expression of IL1B (**a**), CXCL8 (**b**) and PTGS2 (**c**) between OA and healthy groups. Expression levels in dataset GSE143514 between OA and healthy groups (d). Red represents the control group, green represents the healthy group. *: *p* < 0.05.

QRT-PCR results further confirmed that IL1B, CXCL8, and PTGS2 exhibited higher mRNA expression in the control group **(**Fig. 6a and c**)**. In addition, significant differences were observed in the expression levels of biomarkers (IL1B, CXCL8, PTGS2) in the GSE143514 dataset, and the expression patterns were consistent with those observed in the GSE55235 and GSE12021 datasets, as well as with the qRT-PCR validation results. (Fig. 6d)

## Discussion

Several factors, including obesity, metabolic syndrome, age, genetics, sex, joint injury, and notably the gut microbiota, are implicated in the progression of OA^4,15^. Evidence suggests that modulating the gut microbiome holds therapeutic potential for OA, primarily by influencing the gut environment and autophagic flux^9,10,16^. Butyrate, a key energy source for maintaining intestinal homeostasis and function, has recently demonstrated inhibitory effects on OA^14^. However, existing research has primarily focused on butyrate’s role in chondrocyte autophagy and inflammatory cell death, with limited understanding of its impact on gene expression, pathway regulation, and immune-inflammatory responses. This study elucidated the mechanisms and associations between BMRGs and OA, explored the biological processes underlying butyrate regulation in OA, and identified novel therapeutic targets for the disease.

The analysis revealed 38 DE-BMRGs, predominantly enriched in pathways associated with cell proliferation, apoptosis, and oxidative stress responses, such as the PI3 K/AKT/mTOR, HIF- 1α, and TNF signaling pathways, all of which are critical in the onset and progression of inflammatory diseases. Previous studies has explored the regulatory roles of these pathways in OA. The PI3 K/AKT/mTOR pathway is involved in OA cartilage degradation, subchondral bone dysfunction, and synovial inflammation by modulating chondrocyte autophagy, apoptosis, and inflammation^17^. The HIF- 1α pathway regulates OA progression through mechanisms such as mitophagy, ferroptosis, inflammation, and osteoclast differentiation^18^, while the TNF signaling pathway is closely linked to inflammatory OA pain *via*its regulation of mitophagy and oxidative stress^19^. These pathways and processes are deeply integrated into OA pathophysiology, aligning with findings from prior studies.

Six candidate biomarkers, IL1B, IGF1, CXCL8, PTGS2, SERPINE1, and MMP9 were identified through the intersection of signature genes from SVM-RFE and LASSO analysis in this study. Among these, IL1B, CXCL8, PTGS2, and SERPINE1 were downregulated, while IGF1 and MMP9 were upregulated in the OA group. However, after undergoing the rank sum test, IGF1, SERPINE1, and MMP9 showed no significant difference in the validation set. IL1B, CXCL8, and PTGS2 (COX2) were the candidate biomarkers that showed elevated expression in the control group with experimental verification. Although the PCR validation of PTGS 2 expression was not significant, its expression trend in the OA group was consistent with our prediction. Additionally, we sought out the most recent dataset (GSE143514) and also observed significant differences in the expression levels of PTGS2, which could be served as the potential biomarkers for OA. Thus, we speculate that this insignificant outcome may be attributed to experimental error associated with the smaller sample size.

The results confirmed that IL1B, CXCL8, and PTGS2 (COX2) were the key biomarkers for OA with energy metabolism and immune-inflammatory responses. The same conclusion was reached in other studies on OA, especially regarding the widespread participation in inflammation expression in chondrocytes. IL1B, a macrophage-associated gene, is found in both the synovium and chondrocytes, serving as a diagnostic marker for OA with a pro-inflammatory effect^20^. CXCL8, a proinflammatory cytokine in chondrocytes, has been detected within cytokine and chemokine expression profiles in patients with OA^21^. It is also recognized as an age-related gene, detectable in the serum of patients with OA^22^. PTGS2 plays a critical role in inflammatory responses and is closely linked to the activation of inflammatory cells, particularly the polarization of macrophages^23^. NSAIDs, which inhibit COX2 expression in synovial macrophages, have been employed to alleviate OA-related pain^4^. These cytokines—IL1B, CXCL8, and PTGS2 (COX2)—are integral to the inflammatory pathways and are supported by other studies exploring inflammatory mechanisms in OA.

Aberrant expression of IL1B, CXCL8, and PTGS2 enriches OA-related pathways involved in cell proliferation, differentiation, oxidative phosphorylation metabolism, and immune-inflammatory responses. For instance, the MHC II antigen presentation pathway is enhanced by citrullination of vimentin through autophagy of synovial fibroblasts in OA^24^. Clanchy et al.^25^observed differential expression of the MHC class II invariant chain (CD74) p41 isoform by M1-like macrophages, correlating with the mechanistic pathogenesis of human arthritis. In another related metabolic pathway, oxidative phosphorylation is often diverted to glycolysis in OA chondrocytes under stress conditions, enabling metabolism adaptation to microenvironmental, regulated by AMPK and mTOR signaling^26^. Xiang et al.^27^reported that OA progression can be delayed through modulation of oxidative phosphorylation, improving mitochondrial function and promoting anaerobic glycolysis. Additionally, oxidative phosphorylation has been shown to impact mitochondrial function and energy metabolism in OA chondrocytes^28,29^. Moreover, pathways related to cell proliferation and differentiation, immune-inflammatory responses, and glycosaminoglycan degradation, such as the JAK-STAT, MAPK, and NLR pathways, were also found to be enriched, with previous studies linking them to OA progression^15^. In summary, modulation of these signaling pathways could offer promising therapeutic strategies for OA.

Our results confirmed that multiple immune cells contribute to the OA-related degeneration process, with particular emphasis on mast cells, plasma cells, and resting memory CD4 T cells, which exhibited significant expression differences. Notably, activated mast cells emerged as target immune cells, with a significant positive correlation to the biomarkers. Mast cells are multifunctional in the OA synovium, with the infiltration of activated mast cells strongly associated with disease severity and progression^30^. Their pro-inflammatory effects, validated in mouse models, highlight their potential for developing novel animal models to study OA pathogenesis^31^. Additionally, the increased prevalence of mast cells in OA synovial tissue is linked to structural damage, inflammation, and pain, highlighting their involvement in OA pathophysiology^32^. It is thus hypothesized that the identified biomarkers (IL1B, CXCL8, and PTGS2) may contribute to OA development by influencing mast cells infiltration.

The immune regulatory factors interleukin- 6 (IL- 6) and tumor necrosis factor ligand superfamily member 9 (TNFSF9), which were elevated in the OA group, showed a positive correlation with the biomarkers. IL- 6 has been implicated in the pathogenesis of inflammatory diseases and is closely associated with matrix metalloproteinase (MMP) levels in knee synovial fluid, cartilage degradation, and OA severity^33^. Cytokines such as IL- 1β and growth factors like TGF-β stimulate IL- 6 production in human chondrocytes^34^, with IL- 6 being induced *via*NF-κB signaling and promoting PTGS2 (COX 2) expression^35^. Although there are limited reports linking TNFSF9 to OA, our study found that IL- 6 and TNFSF9 levels were positively correlated with biomarkers, suggesting their involvement in OA inflammatory pathways. Furthermore, CD86 and CXCL12, which were detected in synoviocytes, showed a negative correlation with the biomarkers. The CXCL12 signaling axis has gained recognized as a potential therapeutic target in OA. Li et al.^36^ reported that G protein-coupled receptor 4 modulates OA development *via*NF-κB/MAPK signaling by activating the CXCL12/CXCR7 axis. OA pathophysiology involves processes such as chondrocyte degeneration, aggrecanase activation^37^, cell apoptosis, extracellular matrix degradation^38^, synovial macrophage recruitment, and synovitis^39^, all of which are regulated by the CXCL12 axis. Whether positively or negatively regulated by the biomarkers, the IL- 6 and CXCL12 axis-related pathways may offer novel insights into the pathogenic mechanism of OA and provide potential targets for therapeutic development.

IL1B, CXCL8, and PTGS2 (COX 2) are regulated by hsa-miR- 4312, hsa-miR- 4687 - 3p, hsa-miR- 4436b- 5p, and hsa-miR- 6775b- 3p, with all of the lncRNAs regulating CXCL8 through hsa-miR- 1294. These key miRNAs are predicted to play a significant role in improving OA pathophysiology, pending more detailed mechanism investigations. Previous studies have shown that hsa-miR- 1294 acts as a tumor suppressor in several cancers^40^, while hsa-miR- 4687 - 3p holds potential as a diagnostic and carcinogenic biomarker in lung cancer^41^, Additionally, hsa-miR- 4312 has been linked to BAG3 expression, enhancing PDAC metastasis in pancreatic ductal adenocarcinoma^42^. However, the specific mechanisms through which these miRNAs and lncRNAs participate in OA remain underexplored. Several miRNAs, including hsa-miR- 29b, hsa-miR- 30a, and hsa-miR- 125b, have been identified as key regulators of OA through their modulation of PTGS2. Guan et al.^43^reported that exosomal miR- 125 from OA chondrocytes disrupted subchondral bone homeostasis and exacerbated cartilage damage in aging mice. Other studies have highlighted the miR- 29 family as key regulators of OA inhibiting chondrogenic and osteoclast differentiation or fibrosis, providing avenues for therapeutic targeting^44^. Similarly, the miR- 30 family^45^,particularly miR- 30a/b, was found to be upregulated in cartilage tissue from patients with OA, targeting key transcription factors such as RUNX2, SOX9, and beclin- 1, through pathways critical for bone homeostasis, including Wnt/β-Catenin, mTOR, and PI3 K/AKT^45^. These findings emphasize that miRNAs and lncRNAs related to the identified biomarkers play a regulatory role in OA progression, and several of them are newly identified in this study, warranting further exploration.

In the drug prediction analysis, pranoprofen and other agents were identified as potential candidates for OA treatment. Several of these drugs, including pranoprofen^46^, and indomethacin^47^, which target PTGS2 (COX2), have been clinically applied for their anti-inflammatory effects to alleviate pain. However, many of the other drugs identified remain in the experimental phase and require further validation for their efficacy in OA treatment^48^. Zhang et al.^49^ reported that donepezil, an acetylcholinesterase inhibitor used for dementia in Alzheimer’s disease, can prevent collagen II degradation induced by TNF-α, suggesting its potential therapeutic effect in OA. Other compounds, such as rivanicline (RJR- 2403), an α7-nicotinic receptor agonist, have shown promise in Alzheimer’s disease but lack studies in the context of chondrocytes or OA. Thus, while the drugs identified in this study have theoretical potential for OA treatment, they require further experimental verification. Given their connection to butyrate metabolism, drugs used in treating biliary and digestive system diseases may hold promise for OA therapy.

This study has several limitations. Despite the use of machine learning for biomarker screening, identification, and expression validation, a lack of in vitro experimental validation or further clinical confirmation, such as Western blotting, immunohistochemistry, and animal models, remains. Numerous cytokines, immune cells, drugs, and genes involved in butyrate metabolism were implicated in OA, warranting further investigation. Moreover, the sample size utilized for experimental validation was small. This is primarily due to the difficulty in obtaining normal cartilage and securing the subjects’ consent. The small sample size does introduce some deviation in the results of validation studies, which in turn affects their credibility. In future studies, we can enhance the reliability of the results by obtaining more recent and diverse cartilage samples, or by employing advanced techniques such as tissue engineering or organoid models to overcome the limitations of sample acquisition. Additionally, immune imprinting and animal models can also be utilized to provide a more reliable foundation for the early diagnosis and treatment of OA.

Through comprehensive bioinformatics analyses and expression validation, three biomarkers—IL1B, CXCL8, and PTGS2—were identified as associated with butyrate metabolism in OA. Enriched pathways, including those related to cell metabolism, immune responses, immune cell activation, and potential therapeutic agents, were also analyzed. Several metabolic processes, cytokines, and drug, which were underexplored in previous OA research, were identified and reported for the first time. This study illuminates the complex role of butyrate metabolism in modulating immune-inflammatory processes in OA, providing a theoretical foundation and novel targets for future OA therapies.

## Materials and methods

### Data collection

OA related datasets (GSE55235, GSE12021 and GSE143514) (GPL96, HG-U133 A and GPL20795) were retrieved from the Gene Expression Omnibus (GEO) database.

The GSE55235 dataset contained 10 synovial tissue samples from both OA and control groups, while GSE12021 comprised 10 OA and 9 control synovial tissue samples. The GSE143514 dataset contained 5 OA and 3 control synovial tissue samples. Using the GeneCards database (http://www.genecards.org/), 2,183 candidate BMRGs were identified by searching for “butyric acid”. A total of 385 BMRGs, with a “relevance score” ≥ 15, were selected for further analysis.

### Differential expression analysis

DEGs between OA and control groups in the GSE55235 dataset were screened using the limma package (version 3.56.2)^50^with criteria of p.adjust < 0.05 and |log2 FoldChange (FC)| > 1. The top 10 DEGs were visualized using a volcano plot and heatmap generated with the ggplot2 (version 3.4.4)^51^and ComplexHeatmap packages (version 2.16.0)^52^.

### DE-BMRG identification and analysis

The intersection of DEGs and BMRGs was defined as DE-BMRGs. To explore thei potential biological functions, GO and KEGG enrichment analyses were performed using the ClusterProfiler package (version 4.8.3) (p.adjust < 0.05)^53–56^. PPIs of DE-BMRGs were predicted through the STRING database (*p*< 0.05), and the PPI network was visualized using the Cytoscape package (version 3.9.1)^57^. For further hub gene identification, four algorithms (degree, MNC, closeness, and MCC) in the cytoHubba plugin were applied to select candidate hub genes, with the intersections of the top 10 genes identified by each algorithms considered as candidate hub genes.

### Biomarker identification and expression validation

Signature genes were selected using SVM-RFE and LASSO analyses, implemented through the mlbench (version 2.1–3.1) and glmnet package (version 4.1–8)^58^. Candidate biomarkers were obtained by overlapping signature genes derived from both machine-learning algorithms. SVM-RFE was a commonly used classification method that demonstrated several unique advantages in addressing small sample sizes, non-linearity, and high-dimensional pattern recognition. LASSO regression was a statistical method that constructed a penalty function to obtain a more refined model, which compressed certain regression coefficients, reduced dimensionality, and helped avoid multicollinearity and overfitting in multiple regression models. By combining both algorithms, the accuracy of the selection process was further improved, and overfitting was minimized. The expression of candidate biomarkers was compared between OA and control groups using the Wilcoxon test. Genes with consistent expression and significant differences across both GSE55235 and GSE12021 datasets were designated as biomarkers. Correlations between these biomarkers were analyzed using Spearman analysis.

### Gene set enrichment analysis (GSEA) and similarity analysis of biomarkers

To investigate potential related pathways and mechanisms, GSEA was performed. In the GSE55235 dataset, Spearman’s correlation coefficient was calculated between each biomarker and all genes using the psych package (version 2.0.9)^59^. After sorting by correlation coefficient, GSEA was conducted using the ClusterProfiler package (*p*.adjust < 0.05), with the Reactome gene set (c2.cp.reactome.v2023.2.Hs.symbols.gmt) used as the background. Additionally, functional similarity of biomarkers was assessed using the GOSemSim package (version 2.26.1)^60^.

### ANN construction and evaluation

The relationship between each biomarker and the occurrence of OA was investigated using the neuralnet package (version 1.44.2)^61^. An ANN diagnostic model was constructed based on biomarkers from the GSE55235 dataset and a logistic regression model. Model performance was evaluated and verified using receiver operating characteristic (ROC) curves in both the GSE55235 and GSE12021 datasets.

### Gene set variation analysis (GSVA)

To assess differences in KEGG pathway between high- and low-expression groups, GSVA was performed. Samples from the GSE55235 dataset were classified into high- and low-expression groups based on the median biomarker expression levels. GSVA score for KEGG pathways were calculated using the “c2.cp.kegg.v2023.1.Hs.symbols.gmt” gene set. Differences between the groups were analyzed using the Wilcoxon.test (*p* < 0.05).

### Immune infiltration analysis

Immune cell composition in the GSE55235 dataset was determined using the CIBERSORT algorithm based on the Leukocyte Signature Matrix 22 (LM22). Differential immune cell were identified using the Wilcoxon test, and their relationships were visualized. Correlations between biomarkers and differential immune cells were determined through Spearman correlation analysis. To further investigate the relationships between biomarkers and immune regulatory factors, Spearman correlation analysis was performed using a set of 24 immune suppressive factors and 46 immune stimulatory factors derived from published literature^62^.

### Regulatory network construction, disease ontology (DO) analysis, and drug prediction

MiRNAs targeting biomrakers were predicted using the miRDB (target score ≥ 80, http://mirdb.org) and miRWalk (binding point = 1, http://mirwalk.uni-hd.de/) databases. Key miRNAs were identified by intersecting results from both databases. lncRNAs targeting these key miRNAs were then predicted using the miRNet database (https://www.mirnet.ca/). Regulatory networks, including miRNA-lncRNA, miRNA-lncRNA-mRNA, and lncRNA-miRNA-mRNA interactions, were visualized using Cytoscape. Disease Ontology (DO) enrichment analysis was conducted using the DOSE package (version 3.26.2)^63^ (*p*.adjust < 0.05) to identify the association between biomarkers and disease. To explore potential drugs for OA treatment, the DrugBank database (http://www.drugbank.ca) was utilized, searching for candidate drugs based on the identified biomarkers.

### Validation of biomarker expression levels

To further validated the expression of the final biomarker in OA patients, we utilized qRT-PCR to assess its expression at the mRNA level. A total of 5 pairs samples (5 OA and 5 control samples) were collected from participants at the Affiliated Hospital of Qingdao University, with informed consent obtained from all individuals. The study was approved by the Ethics Committee of the Afficiated Hospital of Qingdao University (approval number: QYFY WZLL 27021). The OA samples were collected from individuals who underwent total knee arthroplasty, while the control samples came from young patients (< 30 years old) with acute knee joint trauma requiring arthroscopic surgery.

Total RNA (50 mg) was extracted from the 10 samples using the 1 ml TRIzol reagent (Ambion, USA) following the manufacturer’s instructions. RNA concentration was measured using a NanoPhotometer N50. cDNA synthesis was performed using the SureScript First-strand cDNA synthesis kit, with reverse transcription carried out on a S1000™ Thermal Cycler (Bio-Rad, USA). QPCR assays were conducted using the CFX Connect Real-time Quantitative PCR System (Bio-Rad, USA) under the following conditions: pre-denaturation at 95 °C for 1 min; denaturation at 95 °C for 20 s, annealing at 55 °C for 20 s, and extension at 72 °C for 30 s for 40 cycles. The relative quantification of mRNA was calculated using the 2^−ΔΔCT^ method. The sequences of all primers are listed in **Supplementary Table ****S1**.

Additionally, we performed the analysis using the Wilcoxon test to validate the expression of the final biomarkers between the OA and control groups in the GSE143514 dataset.

### Statistical analysis

Data processing and analysis were performed using R software (version 4.1.3). Statistical significance between two groups was assessed using the Wilcoxon rank-sum test, with a *p*-value < 0.05 considered statistically significant.

## Electronic supplementary material

Below is the link to the electronic supplementary material.

Supplementary Material 1

## Data Availability

The datasets analysed during the current study are available in the [GEO] repository, [https://www.ncbi.nlm.nih.gov/geo/] and [GeneCards] repository, [https://www.genecards.org/].
