# Supplementary material for: Identification and experimental verification of biomarkers related to butyrate metabolism in osteoarthritis
Source: Sci Rep. 2025 Apr 7;15:11884. doi: 10.1038/s41598-025-97346-z (PMC11977226; doi:10.1038/s41598-025-97346-z)
Supplement: Supplementary file 1 — Supplementary Material 1 [file 41598_2025_97346_MOESM1_ESM.docx]

**Supplementary Table S1 The sequences of all primers.**

| **Primers** | **Sequences** |
| --- | --- |
| IL1B F | AGCTACGAATCTCCGACCAC |
| IL1B R | CGTTATCCCATGTGTCGAAGAA |
| CXCL8 F | TGGCACGTCATCGTGTTACC |
| CXCL8 R | GCCAGATCACCTTCCACACA |
| PTGS2 F | TTGCATTCTTTGCCCAGCAC |
| PTGS2 R | ACCGTAGATGCTCAGGGACT |
| GAPDH F | CGAAGGTGGAGTCAACGGATTT |
| GAPDH R | ATGGGTGGAATCATATTGGAAC |
